# Polymicrobial Sepsis Impairs Antigen-Specific Memory CD4 T Cell-Mediated Immunity

**DOI:** 10.3389/fimmu.2020.01786

**Published:** 2020-08-12

**Authors:** Frances V. Sjaastad, Tamara A. Kucaba, Thamotharampillai Dileepan, Whitney Swanson, Cody Dail, Javier Cabrera-Perez, Katherine A. Murphy, Vladimir P. Badovinac, Thomas S. Griffith

**Affiliations:** ^1^Microbiology, Immunology, and Cancer Biology Ph.D. Program, University of Minnesota, Minneapolis, MN, United States; ^2^Department of Urology, University of Minnesota, Minneapolis, MN, United States; ^3^Department of Microbiology and Immunology, University of Minnesota, Minneapolis, MN, United States; ^4^Center for Immunology, University of Minnesota, Minneapolis, MN, United States; ^5^Medical Student Summer Research Program in Infection and Immunity, University of Minnesota, Minneapolis, MN, United States; ^6^Medical Scientist Training Program, University of Minnesota, Minneapolis, MN, United States; ^7^Interdisciplinary Graduate Program in Immunology, University of Iowa, Iowa City, IA, United States; ^8^Department of Pathology, University of Iowa, Iowa City, IA, United States; ^9^Department of Microbiology and Immunology, University of Iowa, Iowa City, IA, United States; ^10^Masonic Cancer Center, University of Minnesota, Minneapolis, MN, United States; ^11^Minneapolis VA Health Care System, Minneapolis, MN, United States

**Keywords:** sepsis, immune suppression, CD4 T cells, memory, IFN-gamma

## Abstract

Patients who survive sepsis display prolonged immune dysfunction and heightened risk of secondary infection. CD4 T cells support a variety of cells required for protective immunity, and perturbations to the CD4 T cell compartment can decrease overall immune system fitness. Using the cecal ligation and puncture (CLP) mouse model of sepsis, we investigated the impact of sepsis on endogenous Ag-specific memory CD4 T cells generated in C57BL/6 (B6) mice infected with attenuated *Listeria monocytogenes* (Lm) expressing the I-A^b^-restricted 2W1S epitope (Lm-2W). The number of 2W1S-specific memory CD4 T cells was significantly reduced on day 2 after sepsis induction, but recovered by day 14. In contrast to the transient numerical change, the 2W1S-specific memory CD4 T cells displayed prolonged functional impairment after sepsis, evidenced by a reduced recall response (proliferation and effector cytokine production) after restimulation with cognate Ag. To define the extent to which the observed functional impairments in the memory CD4 T cells impacts protection to secondary infection, B6 mice were infected with attenuated *Salmonella enterica-*2W (*Se*-2W) 30 days before sham or CLP surgery, and then challenged with virulent *Se-*2W after surgery. Pathogen burden was significantly higher in the CLP-treated mice compared to shams. Similar reductions in functional capacity and protection were noted for the endogenous OVA_323_-specific memory CD4 T cell population in sepsis survivors upon Lm-OVA challenge. Our data collectively show CLP-induced sepsis alters the number and function of Ag-specific memory CD4 T cells, which contributes (in part) to the characteristic long-lasting immunoparalysis seen after sepsis.

## Introduction

The importance of a functional immune system for overall health is dramatically illustrated by individuals with immune system defects being highly susceptible to serious and often life-threatening infections. States of immune deficiency can be congenital (e.g., impaired T and/or B cell development) or acquired [e.g., HIV infection, iatrogenic (post-organ transplant) immune suppression, or surgery/trauma]. Studies interrogating the events leading to acquired immunodeficiency are done with the goal of designing treatment modalities to restore immune system function and reduce the susceptibility to infection.

Sepsis causes millions of deaths annually worldwide (1). Defined as a systemic inflammatory response syndrome during a disseminated infection (2, 3), early stages of sepsis are marked by a potentially fatal hyperinflammatory state driven by proinflammatory cytokines (4–6). Concurrent with this hyperinflammation is system-wide transient loss of multiple immune cell types that decreases the ability of the septic host to respond to the primary infection or secondary nosocomial infection. Advancements in critical care medicine have improved survival rates of patients following the initial sepsis-inducing injury (7–10), where acute death from sepsis is no longer the major cause of mortality for these patients. Currently, ~70% of sepsis-related deaths occur after the first 3 days of the disorder as the result of a secondary infection, with many patient deaths occurring weeks and months later (11). Interestingly, the sepsis-induced lymphopenia is transient, and the once hyperinflammatory immune response transitions to a prolonged immunosuppressive state even though the cellular composition of the immune system numerically returns to normal. In fact, the prolonged immune suppression that develops after a septic event is now considered a leading reason for the extended period of increased susceptibility to pathogens normally handled by the immune system in healthy individuals (11, 12).

CD4 T cells are among the immune cells significantly depleted during the acute stage of sepsis (13), but gradually recover during the immunosuppressive phase (14). CD4 T cells support the function of a variety of immune cells needed to mount a productive and protective immune response (15), and perturbations in the CD4 T cell compartment can dramatically affect overall immune system fitness. The ability to develop and sustain memory cells after infection or immunization is a hallmark of adaptive immunity and basis for protective vaccination against infectious disease (16, 17). Memory CD4 T cells possess several important features that distinguish them from naïve CD4 T cells. First, there are increased numbers of memory CD4 T cells compared to precursors, providing better coverage and a more rapid cellular response during re-challenge. Memory CD4 T cells have experienced cell-intrinsic “programming” changes that allow for rapid expression of effector cytokines, chemokines, and cytotoxic molecules. Additionally, memory CD4 T cells establish residence in both lymphoid and non-lymphoid tissues (18, 19). Finally, the number of memory CD4 T cells present at the end of the contraction phase of a primary response is maintained for the life of the host (20). Maintenance of memory CD4 T cell responses over time is a dynamic process, depending on subsequent encounters with either cognate or non-related Ag/infections that have the potential to change their phenotype and function (15). Similar to the primary response, the magnitude of a memory CD4 T cell response directly correlates with the quantity and quality of memory CD4 T cells present at the time of re-challenge. Thus, changes in composition and function of naive and memory CD4 T cells can result in impaired immunity and increased susceptibility to subsequent infections (21, 22). The present study took advantage of our ability to track the number and function of endogenous Ag-specific memory CD4 T cells in the wake of a septic event [using the cecal ligation and puncture (CLP) model of polymicrobial sepsis]. Our data demonstrate sepsis leads to dramatic and transient decline in pre-existing memory CD4 T cell numbers, with sustained functional impairments, which contribute to the overall increased susceptibility to secondary infections in sepsis survivors.

## Materials and Methods

### Mice

Female C57BL/6 mice (8-weeks old) were purchased from the National Cancer Institute (Frederick, MD). Female pet store mice were purchased from local pet stores in the Minneapolis-St. Paul, MN metropolitan area. All mice were housed in AALAC-approved animal facilities at the University of Minnesota at the appropriate biosafety level (BSL-1/BSL-2 for SPF B6 mice, and BSL-3 for cohoused B6 and pet store mice). SPF B6 and pet store mice were cohoused at a ratio of 8:1 in large rat cages for 60 days to facilitate microbe transfer (23, 24). In all experiments, including those using cohoused mice, mice were age-matched. Experimental procedures were approved by the University of Minnesota Institutional Animal Care and Use Committees and performed following the Office of Laboratory Animal Welfare guidelines and PHS Policy on Human Cancer and Use of Laboratory Animals.

### Cecal Ligation and Puncture (CLP)

Sepsis was induced by CLP (25). Briefly, mice were anesthetized using isoflurane (2.5% gas via inhalation). The abdomen was shaved and disinfected with 5% povidone-iodine antiseptic. Bupivacaine (6 mg/kg s.c.) was then administered at the site where a midline incision was made. The distal third (~1 cm) of the cecum was ligated with 4–0 silk suture and punctured once with a 25-g needle to extrude a small amount of cecal content. After returning the cecum to the abdomen, the peritoneum was closed via continuous suture and the skin was sealed using surgical glue (Vetbond; 3M, St. Paul, MN). Post-operative analgesia and fluid resuscitation occurred at the conclusion of surgery and the following 3 days in the form of meloxicam (2 mg/kg) in 1 ml saline. Mice were monitored daily for weight loss and pain for at least 5 days post-surgery. To control for non-specific changes from the surgery, sham mice underwent the same laparotomy procedure excluding ligation and puncture.

### Experimental Pathogens and Infections

C57BL/6 mice were immunized with attenuated *Listeria monocytogenes*-2W1S (Lm-2W1S) or Lm-OVA (10^7^ CFU i.v.) or attenuated *Salmonella enterica* serovar Typhimurium strain BRD509-2W1S (*Se*-2W1S; AroA^−^; 10^6^ CFU i.v.) 30 days before sham or CLP surgery to generate memory CD4 T cells. In some experiments, mice received a second infection with attenuated Lm-2W1S (10^7^ CFU i.v.), virulent Lm-OVA (10^4^ CFU i.v.), or virulent *Se*-2W1S (10^3^ CFU i.v.). In experiments where mice received a secondary virulent Lm-OVA bacterial challenge, mice were depleted of CD8 T cells by injecting 100 μg anti-CD8 mAb (clone 2.43) i.v. 3, 2, and 1 days prior to secondary infection. In experiments where mice received a secondary virulent *Se*-2W1S bacterial infection, some of the mice were depleted of CD4 T cells by injecting 800 μg of anti-CD4 mAb (clone GK1.5) i.v. 7 days before and 400 μg i.v. 4 and 3 days before challenge. To measure the clearance of the secondary infection of virulent Lm-OVA or virulent *Se*-2W1S, livers and spleens were removed 3 or 7 days post-infection, respectively, placed in 0.2% IGEPAL solution (Sigma-Aldrich), and homogenized. Serial dilutions of the homogenate were plated on tryptic soy broth agar containing 50 μg/ml streptomycin (for Lm-OVA) or 100 μg/ml streptomycin (for *Se*-2W1S), which restricted bacterial growth to the streptomycin-resistant Lm-OVA or *Se*-2W1S used for secondary infection. Bacterial colonies were counted after 24 h incubation at 37°C (26–28).

### Enrichment and Analysis of Ag-Specific CD4 T Cells

I-A^b^-specific tetramers containing 2W1S (EAWGALANWAVDSA) or OVA_323−339_ (ISQAVHAAHAEINEAGR) were used to identify Ag-specific CD4 T cells (29–31). Briefly, I-A^b^ β-chains containing the 2W1S or OVA_323−339_ epitopes covalently linked to the I-A^b^ β-chain were produced in Drosophila melanogaster S2 cells. 2W1S:I-A^b^ or OVA_323−339_:I-A^b^ monomers were then biotinylated and made into tetramers with streptavidin-phycoerythrin (SA-PE; Prozyme). To enrich for Ag-specific CD4 T cells, tetramers (10 nM final concentration) were then added to single cell suspensions in 300 μl tetramer staining buffer (PBS containing 5% FBS, 2mM EDTA, 1:50 normal mouse serum, and 1:100 anti-CD16/32 mAb). The cells were incubated in the dark at room temperature for 1 h, followed by a wash in 10 ml ice cold FACS Buffer. The tetramer-stained cells were then resuspended in 300 μl FACS Buffer, mixed with 25 μl of anti-PE mAb-conjugated magnetic microbeads (StemCell Technologies), and incubated in the dark on ice for 30 min. The cells were washed, resuspended in 3 ml cold FACS Buffer, and passed through an EasySep Magnet (StemCell Technologies) to yield the enriched tetramer positive population. The resulting enriched fractions were stained with a cocktail of fluorochrome-labeled mAb (see below). Cell numbers for each sample were determined using AccuCheck Counting Beads (Invitrogen). Samples were then analyzed using a Fortessa flow cytometer (BD) and FlowJo software (TreeStar Inc., Ashland, OR). The percentage of 2W1S:I-A^b+^ or OVA_323−339_:I-A^b+^ events was multiplied by the total number of cells in the enriched fraction to calculate the total number of 2W1S:I-A^b^- or OVA_323−339_:I-A^b^–specific CD4 T cells.

*In vivo* peptide stimulation was used to determine Ag-specific CD4 T cell cytokine production, as previously described (31–34). Briefly, infected mice were injected i.v. with 100 μg of the 2W1S or OVA_323−339_ peptides (synthesized by Bio-Synthesis, Louisville, TX). After 4 h, spleens were harvested in media containing 10 μg/ml brefeldin A. The resulting cell suspensions were fixed, permeabilized, and stained with anti-IFNγ, -TNF, and -IL-2 mAb.

### Flow Cytometry

To assess the expression of cell surface proteins, cells were incubated with fluorochrome-conjugated mAb at 4°C for 30 min. The cells were then washed with FACS buffer. For some experiments, the cells were then fixed with PBS containing 2% paraformaldehyde. In procedures requiring intracellular staining, cells were permeabilized following surface staining using the transcription factor staining kit (Tonbo), stained for 1 h at 20°C with a second set of fluorochrome-conjugated mAb, and suspended in FACS buffer for acquisition. The fluorochrome-conjugated mAb used in both surface and intracellular stainings were as follows: Dump gate: APC-Cy7 CD11b (clone M1/70; Tonbo), APC-Cy7 CD11c (clone N418; Tonbo), APC-Cy7 B220 (clone RA3-6B2; Tonbo), APC-Cy7 F4/80 (clone BM8.1; Tonbo), Ghost Red 780 viability dye (Tonbo). Surface staining: BV650 CXCR5 (clone L138D7; BioLegend), Brilliant Violet 510 CD44 (clone IM7; BioLegend), redFluo 710 CD44 (clone IM7; Tonbo), Brilliant Violet 711 CD8 (clone 53-6.7; BioLegend), Brilliant Ultra Violet 395 Thy1.2 (clone 53-2.1; BD Biosciences)—used as an alternative to CD3 for gating T cells, Brilliant Ultra Violet 496 CD4 (clone GK1.5; BD Biosciences), Alexa Fluor 647 CD49d (clone R1-2; BD Biosciences), FITC CD11a (clone M17/4; eBioscience), PE-Cy7 CD11a (clone M17/4; eBioscience). Intracellular staining: Alexa Fluor 488 Foxp3 (clone FJK-15S; Invitrogen), PE-Cy7 Tbet (clone 4B10; BioLegend), PE Bcl6 (clone K112-91; BD Biosciences), BV650 IFN-γ (clone XMG1.2; BD Biosciences), APC IFN-γ (clone XMG1.2; eBioscience), PE-Cy7 IL-2 (clone JES6-5H4; BioLegend), APC TNF-α (clone MP6-XT22; BioLegend), PE TNF-α (clone MP6-XT22; BioLegend), PE-Cy7 IL-2 (clone JES6-5H4; BioLegend). Gating and fluorescence thresholds were determined using fluorescence minus one (FMO) controls.

### Statistical Analyses

Data shown are presented as mean values ± SEM. GraphPad Prism 8 was used for statistical analysis, where statistical significance was determined using two-tailed Student *t*-test (for 2 individual groups, if unequal variance Mann-Whitney *U*-test was used) or group-wise, one-way ANOVA analyses followed by multiple-testing correction using the Holm-Sidak method, with α = 0.05. ^*^p < 0.05, ^**^p < 0.01, ^***^p < 0.005, and ^****^p < 0.001.

## Results

### The Number of Pre-existing Memory CD4 T Cells Fluctuate After Sepsis

**Figure 1:**
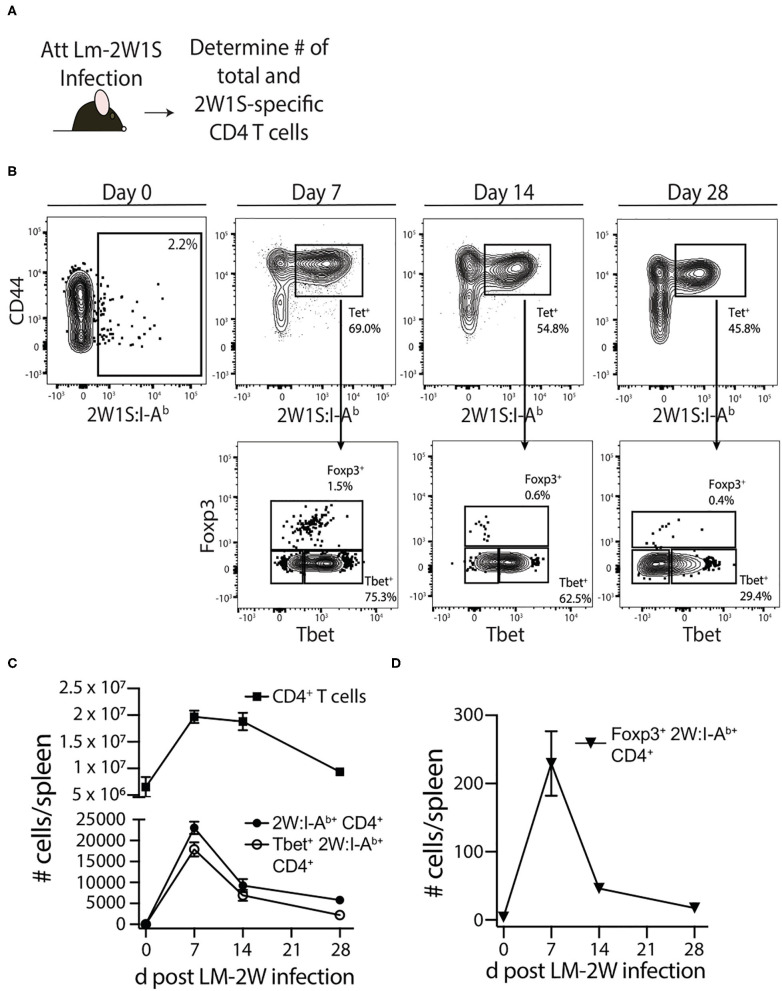
Generation of Ag-specific memory CD4 T cells following attenuated *Listeria monocytogenes*-2W1S infection. **(A)** Experimental design—B6 mice were immunized with attenuated *Listeria monocytogenes*-2W1S (10^7^ CFU i.v.). The number of 2W1S-specific CD4 T cells were determined before and after infection. **(B)** Representative flow plots show the gating strategy used to identify 2W1S:I-A^b+^ cells first identified as being CD3^+^ and CD4^+^. From the tetramer^+^ gate, cells expressing the transcription factors Tbet (Th1 phenotype) or Foxp3 (regulatory T cell phenotype) were then identified. Positive and negative gating determined using FMO controls. The number of **(C)** total CD4 T cells, 2W1S-specific CD4 T cells, Tbet^+^ 2W1S-specific CD4 T cells, and **(D)** Foxp3^+^ 2W1S-specific CD4 T cells in the spleen was determined 7, 14, and 28 days after attenuated LM-2W1S infection. Data shown are representative from 3 independent experiments, with at least 3 mice/group/time point in each experiment.

Septic patients have reduced delayed-type hypersensitivity (DTH) responses, marked by a failure to respond to skin testing with Ag to which previous exposure is known to have occurred (35–37). DTH responses are driven in large part by memory CD4 T cells—even though other immune cells such as CD8 T cells and antigen presenting cells (APCs) participate in the response—and DTH can be used as an assessment of overall immune system fitness (38). To more directly and rigorously interrogate the long-term consequences of sepsis on memory CD4 T cells, we used a protocol where an endogenous, Ag-specific memory CD4 T cell population was generated by infection with attenuated *Listeria monocytogenes* engineered to express the I-A^b^-restricted peptide 2W1S (Lm-2W1S) 30 days before performing sham/CLP surgery (Figure 1A). We also employed a peptide:MHC II (I-A^b^) tetramer-based approach to identify the endogenous 2W1S-specific CD4 T cells before and after sham/CLP surgery. Initially, spleens were harvested from naïve mice and mice at 7, 14, and 28 days post-infection to document the expansion, contraction, and establishment of memory 2W1S-specific CD4 T cells (Figure 1B). The majority of memory 2W1S-specific CD4 T cells adopted a Th1 (Tbet^+^) phenotype (Figure 1C), but some cells upregulated Foxp3 suggesting their differentiation into regulatory T cells (Figure 1D).

**Figure 2:**
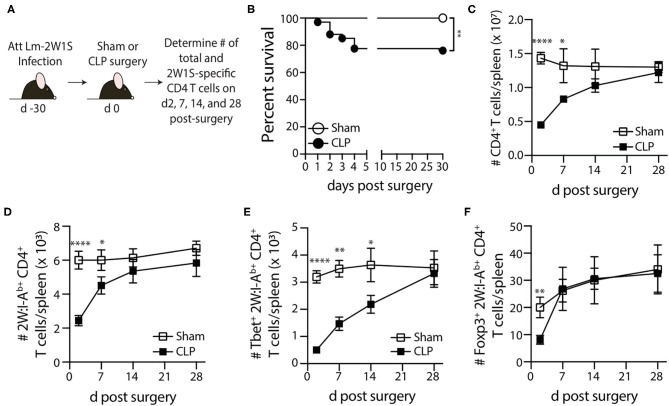
Loss and recovery of 2W1S-specific memory CD4 T cells following CLP-induced sepsis. **(A)** Experimental design—B6 mice were immunized with attenuated *Listeria monocytogenes*-2W1S (10^7^ CFU i.v.) 30 days before sham or CLP surgery. The number of 2W1S-specific CD4 T cells were determined after surgery. **(B)** Survival of LM-2W-infected B6 mice after sham and CLP surgery (*n* = 29 sham; *n* = 67 CLP). The number of **(C)** total CD4 T cells and **(D)** 2W1S-specific CD4 T cells in the spleen was determined 2, 7, 14, and 28 days after sham or CLP surgery by flow cytometry. In addition, the 2W1S-specific CD4 T cells were subtyped based on **(E)** Tbet (Th1 phenotype) and **(F)** Foxp3 (regulatory T cell phenotype) expression. Data shown are cumulative from 3 independent experiments, with at least 3 mice/group/time point in each experiment. *p ≤ 0.05, **p ≤ 0.01, ***p ≤ 0.005, and ****p ≤ 0.001.

Sham or CLP surgery was performed on the remaining mice 30 days post-infection, and the number of total and 2W1S-specific CD4 T cells in the spleen were determined 2, 7, 14, and 28 days post-surgery by flow cytometry (Figure 2A). Our version of the CLP model results in ~20% mortality within the first 4 days after surgery (Figure 2B). Despite this low overall mortality, the mice that underwent CLP show dramatic reductions in number of total CD4 T cells and 2W1S-specific memory CD4 T cells at 2 days post-surgery that gradually recovered by day 30 (Figures 2C,D), which is consistent with our previous data (39–42). Interestingly, the numerical recovery of the Foxp3^+^ 2W1S-specific memory CD4 T cells occurred by day 7 post-CLP, while it took longer for the number of Tbet^+^ 2W1S-specific memory CD4 T cells to return to sham levels (Figures 2E,F). Collectively, these data show pre-existing memory CD4 T cells experience a transient reduction in number during CLP-induced sepsis.

### Loss and Recovery of Ag-Experienced Memory CD4 T Cells in Septic “Dirty” Mice

**Figure 3:**
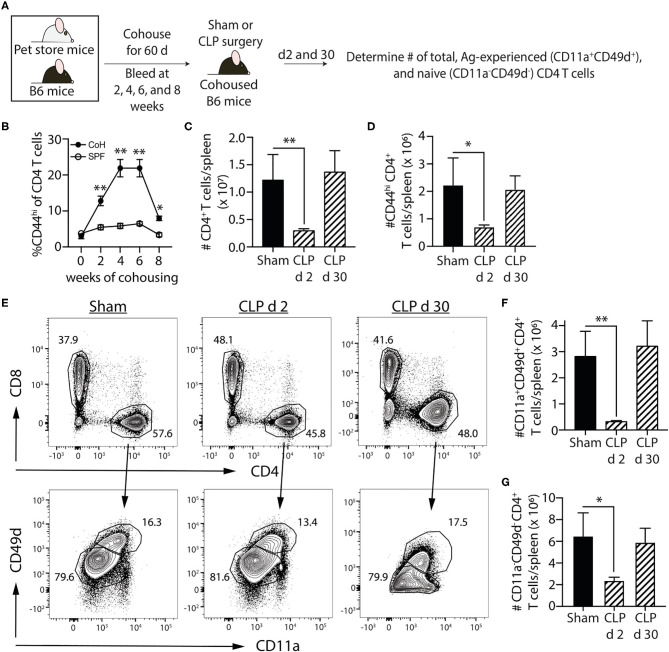
Sepsis induces transient loss in number of pre-existing “Ag-experienced” CD4 T cells in microbially-experienced “dirty” mice. **(A)** Experimental design—SPF B6 mice were cohoused with pet store mice for 60 days to permit microbe transfer and immune system maturation. **(B)** Age-matched SPF and cohoused (CoH) mice were bled prior to and at 2, 4, 6, and 8 weeks after cohousing to determine the frequency of CD44^hi^ CD4 T cells. **(C–G)** Sham or CLP surgery was performed on cohoused B6 mice. The number of total, CD44^hi^, CD11a^+^CD49d^+^ “Ag-experienced,” and CD11a^−^CD49d^−^ naive CD4 T cells in the spleen was determined 2 and 30 days post-surgery by flow cytometry. **(E)** Representative flow plots show gating strategy. Positive and negative gating determined using FMO controls. The number of **(C)** total, **(D)** CD11a^+^CD49d^+^, and **(E)** CD11a^−^CD49d^−^ CD4 T cells was determined. Data shown are representative of 2 independent experiments, with at least 4 mice/group in each experiment. *p ≤ 0.05 and **p ≤ 0.01.

Most preclinical sepsis research done to date has used specific pathogen-free (SPF) mice, which possess an immune system equivalent to that of neonatal humans (23). The vaccinations and infections experienced over a lifetime shape the immune system so rapid and protective functional responses can occur during new microbial encounters. Recently, we investigated the effect of sepsis on standard SPF B6 mice cohoused for 60 days with microbially-experienced “dirty” pet store mice (24). Cohousing laboratory SPF mice with pet store mice permits physiological pathogen transfer and matures the murine immune system to more closely resemble that seen in adult humans (23). To determine the effect of sepsis on multiple memory CD4 T cell populations generated following environmental pathogen/commensal exposure, we performed sham or CLP surgery on cohoused B6 mice age-matched to their SPF counterparts (Figure 3A). Cohousing increases the frequency of circulating memory CD44^hi^ CD4 T cells (compared to age-matched SPF mice; Figure 3B), and the number of total CD4 T cells and CD44^hi^ memory CD4 T cells dramatically decline 2 days after CLP surgery (Figures 3C,D). We extended this analysis of the memory CD4 T cell compartment using a second, more-stringent phenotyping to identify true “Ag-experienced” memory CD4 T cells based on the upregulation of CD11a and CD49d (43). The cohoused mice showed a significant reduction in number of Ag-experienced (CD11^+^CD4d^+^) and naïve (CD11a^−^CD49d^−^) CD4 T cells in the spleen 2 days after CLP that returned to sham levels by day 30 (Figures 3B–E), indicating entire CD4 T cell compartment is susceptible to sepsis-induced numerical reduction. Thus, the data in Figures 2, 3 collectively show memory CD4 cells initially elicited by infection undergo a significant, but transient, numerical reduction in secondary lymphoid organs following CLP-induced sepsis.

### Recall Response to Cognate Ag by Pre-existing Memory CD4 T Cells Is Reduced After Sepsis

**Figure 4:**
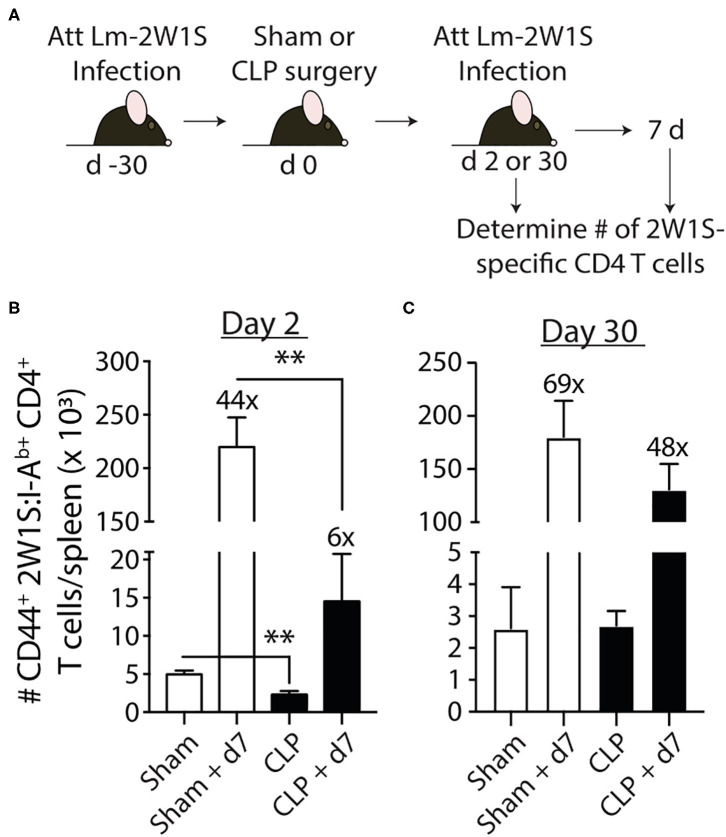
Sepsis impairs the recall response by pre-existing 2W1S-specific memory CD4 T cells to cognate Ag. **(A)** Experimental design—B6 mice were immunized with attenuated *L. monocytogenes*-2W1S (10^7^ CFU i.v.) 30 days before sham or CLP surgery. The mice were given a second infection with attenuated *L. monocytogenes*-2W1S (10^7^ CFU i.v.) **(B)** 2 or **(C)** 30 days after surgery. Total number of 2W1S-specific CD4 T cells in the spleen was determined the day of and 7 days after the second LM-2W1S infection (10^7^ CFU i.v.). The fold increase in cell numbers at day 7 post-secondary infection is indicated. Data shown are representative of 2 independent experiments, with 4 mice/group in each experiment. **p ≤ 0.01.

Data in Figure 2 show 2W1S-specific memory CD4 T cells numerically recover by day 30 post-sepsis. This result would suggest this population of Ag-specific memory CD4 T cells has returned to normal. However, the ability of pre-existing memory CD4 T cells to proliferate, accumulate, and exert effector functions after a second encounter with cognate Ag in the post-septic host has not been rigorously defined. Thus, we first examined the ability of this population of Ag-specific CD4 T cells to proliferate in response to cognate Ag recognition during secondary pathogen encounter. To do this, B6 mice were immunized with attenuated Lm-2W1S 30 days before sham or CLP surgery. The mice were then infected a second time with attenuated Lm-2W1S on day 2 or 30 after surgery. Total numbers of 2W1S-specific CD4 T cells in the spleen were determined before and after the second Lm-2W1S infection (Figure 4A). When infected on day 2 post-surgery, the 2W1S-specific CD4 T cells had significantly reduced proliferative capacity compared to sham-treated mice (Figure 4B). Specifically, the number of 2W1S-specific CD4 T cells in the sham-treated mice expanded 44-fold during the 7 days after secondary infection, but only 6-fold in the CLP-treated mice. However, when challenged on day 30 post-surgery, the proliferative capacity of the 2W1S-specific memory CD4 T cells in CLP-treated mice had nearly recovered to what was found in sham mice (Figure 4C). These data indicate the ability of Ag-specific memory CD4 T cells to proliferate during a recall response to cognate Ag (such as during a secondary infection) is only transiently reduced following sepsis.

**Figure 5:**
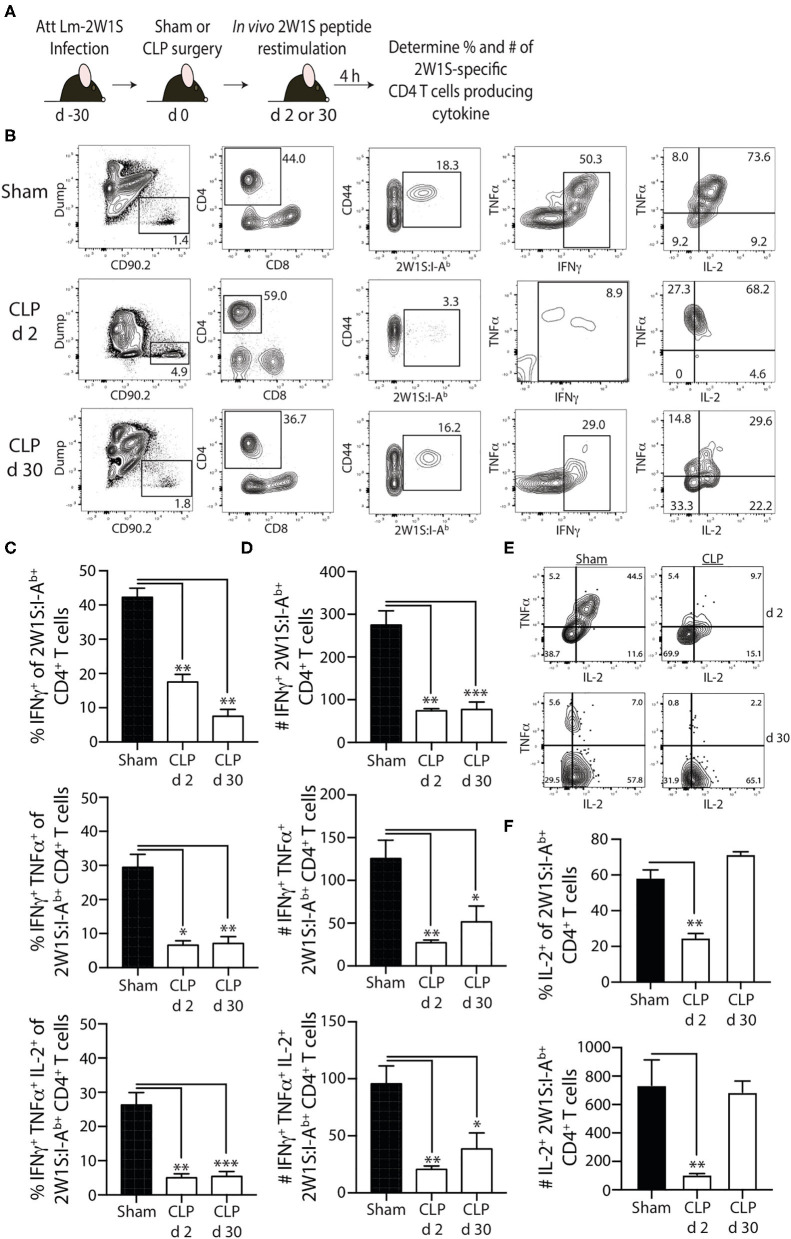
Sepsis impairs the ability of 2W1S-specific memory CD4 T cells to produce effector cytokines after *in vivo* cognate Ag restimulation. **(A)** Experimental design—B6 mice were immunized with attenuated *L. monocytogenes*-2W1S (10^7^ CFU i.v.) 30 days before sham or CLP surgery. Mice were injected with 2W1S peptide (100 μg i.v.) 2 or 30 days after surgery to restimulate the 2W1S-specific memory CD4 T cells. Spleens were harvested 4 h later, and the frequency and number of IFNγ^+^, IFNγ^+^TNFα^+^, and IFNγ^+^TNFα^+^IL-2^+^ 2W1S-specific CD4 T cells was determined by flow cytometry. **(B)** Representative flow plots of intracellular IFNγ, TNFα, and IL-2 detection in CD44^hi^2W1S:I-A^b+^ CD4 T cells after *in vivo* peptide restimulation. Plots show cells gated from 2W:I-A^b^-enriched CD4 T cells from sham- or CLP-treated mice. Positive and negative gating determined using FMO controls. Frequency **(C)** and number **(D)** of CD44^hi^2W1S:I-A^b+^-specific CD4 T cells in the spleen producing IFNγ, IFNγ/TNFα, and IFNγ/TNFα/IL-2. **(E)** Representative flow plots of intracellular IL-2 detection in CD44^hi^2W1S:I-A^b+^ CD4 T cells after *in vivo* peptide restimulation. Plots show cells gated from 2W:I-A^b^-enriched CD4 T cells from sham- or CLP-treated mice. Positive and negative gating determined using FMO controls. **(F)** Frequency and number of CD44^hi^2W1S:I-A^b+^-specific CD4 T cells in the spleen producing IL-2. Data shown are representative of 2 independent experiments, with at least 4 mice/group in each experiment. *p ≤ 0.05, **p ≤ 0.01, and ***p ≤ 0.005.

To examine the effector function of the 2W1S-specific memory CD4 T cells at 2 and 30 days post-surgery, we used *in vivo* peptide restimulation where the Lm-2W1S-immune mice were injected i.v. with 2W1S peptide (27, 31–34). This technique permits the evaluation of cytokine production by Ag-specific (tetramer^+^) CD4 T cells with almost no background staining. Spleens were harvested 4 h after 2W1S peptide injection and processed for flow cytometry to determine the frequency and number of cytokine-producing 2W1S-specific memory CD4 T cells. Lm infection primarily generates a Th1 response (44), therefore we identified the 2W1S-specific CD4 T cells making IFNγ, TNF, and IL-2 (Figures 5A,B). It is important to note the gating strategy employed permits the identification and analysis of bona fide memory CD4 T cells using 2W1S:I-A^b^ tetramers that helps us determine the “per cell” capacity of those cells to produce cytokines. Similar to what was observed with the reduction in proliferative capacity seen 2 days post-CLP-induced sepsis, the frequency and number of single or multi-cytokine producing 2W1S-specific memory CD4 T cells (IFNγ^+^-, IFNγ^+^TNF^+^-, and IFNγ^+^TNF^+^IL-2^+^) was significantly reduced at 2 days post-surgery (Figures 5C,D) and remained reduced 30 days after CLP. Interestingly, when we looked at the 2W1S-specific memory CD4 T cells only making IL-2 30 days after CLP surgery, the frequency and number of IL-2^+^ 2W1S-specific memory CD4 T cells was similar to that seen in sham-treated mice (Figures 5E,F). This data is consistent with that in Figure 4, where we saw a restoration in proliferative capacity at day 30 post-CLP. Together, these data indicate prolonged impairment in the ability of the 2W1S-specific memory CD4 T cells to produce cytokines critical for pathogen clearing immunity when re-stimulated in an Ag-specific manner, despite recovery of cell numbers, and proliferative capacity (by day 30).

### Sepsis Impairs Memory CD4 T Cell-Mediated Immunity to Infection

**Figure 6:**
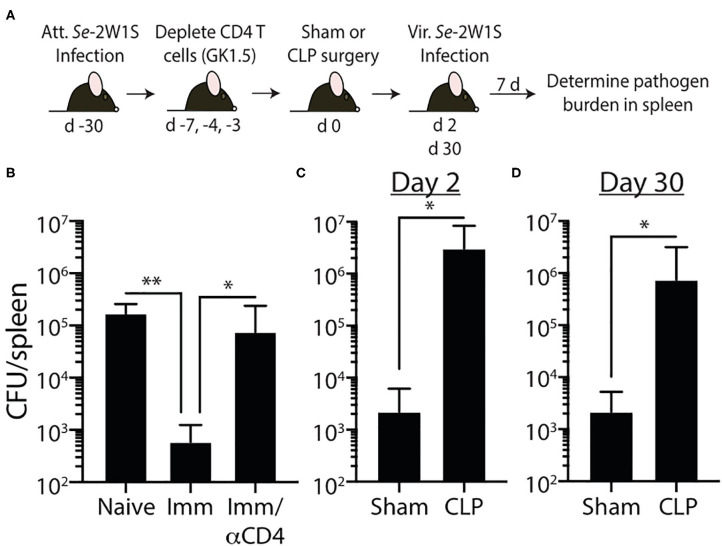
Impaired 2W1S-specific memory CD4 T cell-mediated immunity to secondary *Salmonella*-2W1S infection after CLP-induced sepsis. **(A)** Experimental design—B6 mice were immunized with attenuated *Salmonella enterica* strain BRD509-2W1S (*Se*-2W1S; AroA^−^; 10^6^ CFU i.v.) 30 d before sham or CLP surgery. **(B)** One group of mice (Imm/αCD4) was depleted of CD4 T cells by injecting anti-CD4 mAb GK1.5 i.v. (800 μg on day -7, and 400 μg on days −4 and −3) before second infection with virulent *Salmonella*-2W1S (10^3^ CFU i.v.). Bacterial titers in the spleen were determined 7 days later. **(C–D)** In separate cohorts of attenuated *Se*-2W1S infected mice, sham or CLP surgery was performed. These mice were then challenged with virulent *Se*-2W1S (10^3^ CFU i.v.) **(C)** 2 or **(D)** 30 days after surgery. Bacterial titers in the spleen were determined 7 days later. Data shown are representative of 3 independent experiments, with at least 5 mice/group in each experiment. *p ≤ 0.05, **p ≤ 0.01.

Our data show 2W1S-specific memory CD4 T cells numerically recover by 30 days after CLP surgery, but their ability to produce effector cytokines after re-stimulation remains blunted, suggesting a potential lesion in protective capacity following re-infection. To test this, we wanted to challenge post-septic mice with a virulent pathogen. Our experiments thus far have used attenuated Lm-2W1S to generate a trackable population of endogenous memory Ag-specific CD4 T cells; however, no virulent form of Lm-2W1S exists. Therefore, in the following experiments we employed two different murine models of infection using virulent bacterial strains. It is important to note that while the bacteria strains selected are pathogens not typically found in human septic patients, their use as experimental pathogens is well-established and modification to express known I-A^b^-restricted epitopes allow us to examine distinct, Ag-specific CD4 T cell responses after CLP. In the first model, we generated 2W1S-specific memory CD4 T cells by infecting mice with attenuated *Salmonella enterica* engineered to express the 2W1S epitope (*Se*-2W). *Salmonella-*2W1S infection stimulates a robust Ag-specific CD4 T cell response (33, 45) because the bacteria replicate in the phagosomes of dendritic cells and macrophages—the location of peptide:MHC II complex formation (28, 46–49). Moreover, mice immunized with *Salmonella*-2W1S demonstrate protective immunity to a secondary infection with virulent *Salmonella* (45). To confirm the importance of *Salmonella*-specific memory CD4 T cells in the protection to virulent *Salmonella* infection, some of the *Se*-2W-infected mice were depleted of CD4 T cells (using the anti-CD4 mAb GK1.5) prior to a second infection of virulent *Se*-2W (Figure 6A). CD4 T cell-replete mice infected with attenuated *Se*-2W had significantly lower pathogen burdens after secondary virulent *Se*-2W infection compared to the CD4 T cell-depleted mice (Figure 6B), and had pathogen burdens comparable to that seen in naïve mice only infected with virulent *Se*-2W. We then performed sham or CLP surgery on a separate cohort of mice infected with attenuated *Se*-2W 30 days prior to surgery. On days 2 or 30 after surgery, all groups were infected with virulent *Se*-2W. Splenic bacterial titers were determined 7 days after virulent *Se*-2W infection, revealing higher burdens in the CLP-treated mice regardless of early or late secondary challenge (Figures 6C,D).

**Figure 7:**
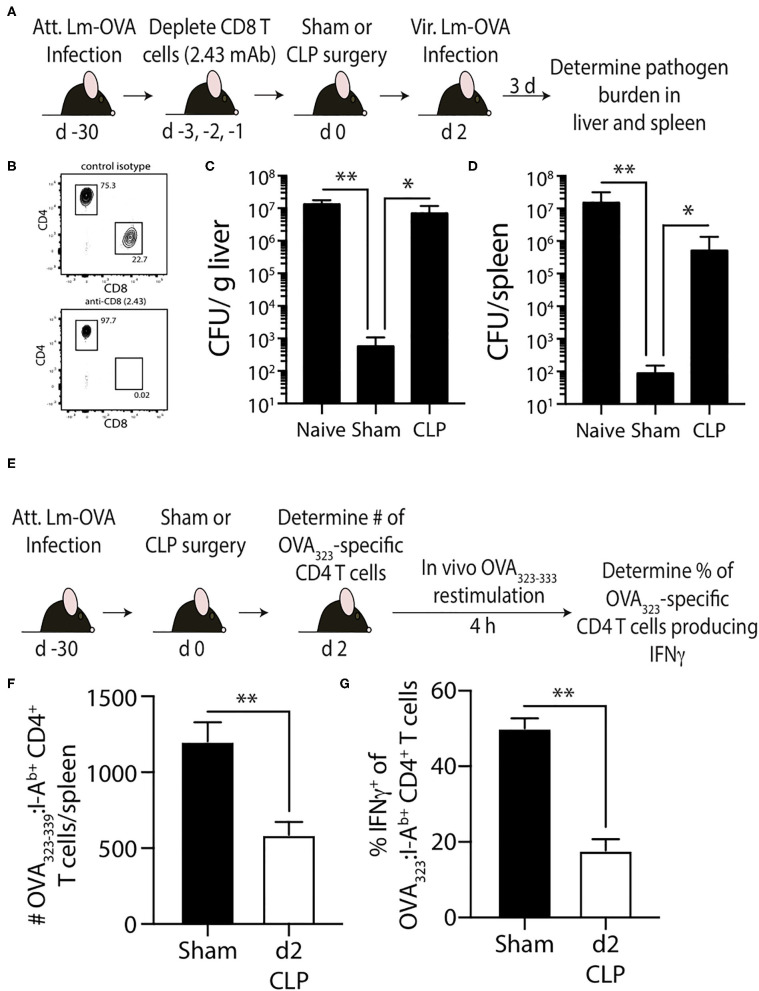
Effect of sepsis on OVA_323−339_-specific memory CD4 T cells. **(A)** Experimental design—B6 mice were infected with attenuated *L. monocytogenes*-OVA (LM-OVA; 10^7^ CFU i.v.) 30 d before sham or CLP surgery. Mice in the naïve, sham, and CLP groups were depleted of CD8 T cells by injecting 100 μg anti-CD8 mAb (clone 2.43) i.v. 3, 2, and 1 days prior to surgery. **(B)** A small amount of blood was collected from the anti-CD8 mAb-treated mice on the day of surgery and staining for CD4 and CD8 T cells. Representative flow plots show the extent of CD8 T cell depletion compared to a reference mouse injected with a control isotype mAb. **(C,D)** The mice were infected with virulent LM-OVA (10^4^ CFU i.v.) 2 days after surgery. Bacterial titers in the liver and spleen were determined 3 days post-vir LM-OVA infection. Data shown are representative of 2 independent experiments, with at least 5 mice/group in each experiment. *p ≤ 0.05 and **p < 0.01. **(E)** Experimental design—B6 mice were infected with attenuated *L. monocytogenes*-OVA (LM-OVA; 10^7^ CFU i.v.) 30 d before sham or CLP surgery. **(F)** On day 2 post-surgery, the number of OVA_323−339_-specific memory CD4 T cells in the spleen were determined. **(G)** A separate cohort of mice were injected with OVA_323−339_ peptide (100 μg i.v.) 2 days after surgery to restimulate the OVA_323−339_-specific memory CD4 T cells. Spleens were harvested 4 h later, and the frequency of IFNγ^+^ OVA_323−339_-specific CD4 T cells was determined by flow cytometry. Data shown are representative of 2 independent experiments, with at least 5 mice/group in each experiment. **p < 0.01.

In the second model, naïve mice were infected with attenuated Lm engineered to express OVA (Lm-OVA) to elicit OVA_323_-specific memory CD4 T cells (50). We tested the ability of the OVA_323_-specific memory CD4 T cells to provide protection against a secondary Lm-OVA infection after sepsis induction. The adaptive immune system does not confer protection against primary Lm infections after CLP (51). CD8 T cells play prominent roles in controlling and eradicating secondary infections by intracellular pathogens that mainly localize to the cytosol of the infected cell, such as *L. monocytogenes* (52), due to the efficient production of peptide:MHC I complexes by the infected cell. However, Lm-specific CD4 T cells can provide sufficient protection to infection even in the absence of CD8 T cells (53–55). Thus, to focus on the memory CD4 T cell-mediated clearance of Lm-OVA, CD8 T cells were depleted using anti-CD8 mAb (clone 2.43) prior to performing sham of CLP surgery (Figures 7A,B). Two days later the mice were infected with virulent Lm-OVA, after which pathogen burden in the liver and spleen was determined. A separate group of naïve, CD8-depleted mice were infected with virulent Lm-OVA for reference. As expected, we saw substantially reduced Lm-OVA burden in both the liver and spleen compared to infected naïve mice (Figures 7C,D). In contrast, the Lm-OVA burdens were dramatically higher in the CLP-treated mice compared to sham mice. To better understand the cause for this reduced protection, we examined the impact of sepsis on the number and function of the OVA_323_-specific memory CD4 T cells (Figure 7E). Just as seen with the 2W1S-specific memory CD4 T cells, CLP-induced sepsis led to a significant reduction in OVA_323_-specific memory CD4 T cell numbers and ability to produce IFNγ after *in vivo* restimulation (Figures 7F,G). Together, the data in Figures 5, 6 show the sepsis-induced long-lasting changes in Ag-specific memory CD4 T cell pools that ultimately impact the ability of the host to properly respond to pathogen re-infection.

## Discussion

Sepsis causes millions of deaths annually (1, 56, 57), and the incidence of sepsis has increased dramatically in recent decades. Understanding the cellular mechanisms that contribute to sepsis-induced immunosuppression is critical for developing effective therapies and improving the survival and quality of life for septic patients. CD4 T cells have the unique flexibility of functioning in an array of immunological settings due to their ability to differentiate into a variety of phenotypic subsets based on the inflammatory milieu produced at the time of primary Ag encounter (58, 59). Clinical data show considerable reduction in number of circulating CD4 T cells (along with other lymphocyte populations) in sepsis patients of all ages (13, 60–62) and at the time of high pathogen burden (63, 64). Moreover, reports of decreased effector CD4 T cell function in critically ill sepsis patients date back to the 1970's with data showing impaired DTH reactions (35). These observations and the fact that DTH is mediated in large part by CD4 T cells (65) bring into question to what extent memory CD4 T cells are numerically and functionally affected by sepsis. However, most (if not all) of the previous studies examined the effect of sepsis on the CD4 T cell compartment in sum, which has the potential to mask some of the unique characteristics of individual Ag-specific populations (34). In contrast to these previous publications, the present study took advantage of pathogens engineered to express defined CD4 T cell epitopes to stimulate the generation of bona fide memory CD4 T cells and reagents to identify and quantitatively and qualitatively evaluate endogenous Ag-specific memory CD4 T cells that have experienced a septic event. As a complement to the experiments using conventional laboratory mice infected with a known experimental pathogen to generate Ag-specific memory CD4 T cells, our “dirty” mouse model allowed us to investigate how multiple populations of memory CD4 T cells are affected during sepsis in an animal with a more adult human-like immune system (23, 24, 66). Together, the experimental model systems used provided a unique means by which sepsis-induced immunoparalysis of memory CD4 T cells was evaluated.

One area of sepsis research that has received considerable attention recently deals with the idea that sepsis may differentially affect naïve and memory T cells. Indeed, there is data to suggest memory CD8 T cells are more resistant to radiation-induced apoptosis than naive cells (67). Cellular apoptotic mechanisms induced by extrinsic (i.e., death receptor) and intrinsic (i.e., mitochondrial) pathways have been suggested to be major contributors to the numerical reduction in total CD4 and CD8 T cells following sepsis (68), but the definitive molecule responsible for initiating lymphocyte apoptosis during sepsis has yet to be indentified. Regardless of the mechanism by which sepsis-induced lymphopenia occurs, studies from a number of laboratories using proven experimental infection models for eliciting Ag-specific memory T cells indicate circulating Ag-experienced memory CD8 T cells are equally susceptible to sepsis-induced attrition as naïve CD8 T cells (26, 69–71). In addition, the circulating memory CD8 T cells exhibit profound impairment in effector functionality (e.g., decreased Ag sensitivity, proliferative capacity, cytokine production, and inability to clear secondary infections) following a septic event (72). The numerical and functional decrease in circulating memory CD8 T cells is not, interestingly, reciprocated in tissue-resident memory CD8 T cells after a moderate sepsis insult that leads to <10% mortality (71). The number of tissue-resident memory CD8 T cells is maintained after sepsis, as well as their ability to produce effector cytokine after re-stimulation. In contrast to the aforementioned similar attrition of naïve and memory CD8 T cells after sepsis, it was recently suggested by Xie et al. that CD44^hi^ CD8 T cells in “memory mice” (generated via *Listeria* and LCMV infection) exhibited significant attrition after CLP while this was not the case for naïve CD44^lo^ CD8 T cells (73). It was surprising to see that CLP sepsis did not lead to a reduction in CD44^lo^ CD8 T cells, as such a reduction has been noted in other papers (14, 39, 40, 74–77). Moreover, these authors examined the bulk CD8 T cell compartment, even though peptide:MHC I tetramers were available to identify LCMV-specific CD8 T cells. The authors also detected increased expression of CD25, PD-1, and 2B4 on the memory CD8 T cells, but did not perform any studies to directly determine if and/or how these proteins may be altering T cell sensitivity to sepsis-induced attention. Needless to say, additional data is needed to conclusively determine the extent of naïve and memory CD8 T cells sensitivity to CLP-induced apoptosis and the potential role played by intrinsic factors (e.g., CD25, PD-1, 2B4, and other proteins) in regulating this sensitivity.

Fewer studies have examined the effect of sepsis on Ag-experienced memory CD4 T cells compared to what has been done for memory CD8 T cells, driving our interest in the current set of experiments. Contrary to the increased susceptibility of memory CD44^hi^ CD8 T cells (vs. naïve CD44^lo^ CD8 T cells) to sepsis-induced apoptosis suggested by Xie et al. (73), data presented by these authors suggested CD44^hi^ CD4 T cells were not more sensitive to attrition during sepsis compared to CD44^lo^ CD4 T cells. As with their CD8 T cell data, the CD4 T cells were examined at the bulk (non-Ag-specific) level. Some of the data presented herein are consistent with the data by Xie et al., but there are some important differences in our study that are worth noting. First, our data show CLP-induced sepsis results in a transient numerical reduction of 2W1S-specific memory CD4 T cells (current study), while 2W1S-specific naive CD4 T cells suffer from prolonged numerical reduction (34). Second, while the numerical reduction was transient, the inability of the 2W1S-specific memory CD4 T cells to produce cytokines upon peptide restimulation was evident out to day 30 post-CLP, similar to what was observed in naïve cells (34). Cytokine “help” from CD4 T cells is a hallmark of this population of immune cells, and the prolonged dysfunction in cytokine production may contribute the generalized immunoparalysis seen during sepsis. Furthermore, the *in vivo* peptide restimulation assay used is a more physiological way of activating the desired Ag-specific population via MHC II presentation of peptide Ag (31–34). Third, despite their reduced ability to produce cytokines at day 30-post-sepsis, 2W1S-specific memory CD4 T cells were surprisingly able to expand upon Ag re-encounter 30 days after CLP to nearly sham levels. It is important to note that the accumulation/expansion of Ag-specific effector CD4 T cells upon Ag re-encounter is dependent on the rate of proliferation (leading to an increase in cell accumulation) and rate of death (decrease in accumulation). We did not measure either of these parameters; however, impairment is clearly seen in day 2 septic mice undergoing secondary challenge since the fold-expansion in numbers from pre-challenge level was significantly diminished (6x compared to 44x—see Figure 4). It is reasonable to suggest that the reduction in accumulation of memory CD4 T cells following a second encounter with cognate Ag to be due to decreased per-cell proliferation, increased death, or both. Together, our data show the 2W1S-specific memory population is similarly prone to the initial sepsis-induced depletion and prolonged inability to produce important inflammatory cytokines compared to their naïve counterparts. However, unlike naïve 2W1S-specific CD4 T cells, Ag-experienced memory 2W1S-specific CD4 T cells quickly recover numerically, as well as their ability to proliferate in response to a secondary infection. It remains to be determined how sepsis affects the number and function of tissue-resident memory CD4 T cells. Moreover, the different recovery rates between the memory and regulatory Foxp3^+^ CD4 T cell populations could suggest additional time-dependent mechanisms that control the ability of sepsis survivors to respond to secondary infection. There have been a few reports specifically looking at the role of regulatory Foxp3^+^ CD4 T cells in sepsis (78–80), and while these data hint at the participation of this CD4 T cell subset in sepsis-induced immune suppression additional evaluation is needed to better define how regulatory CD4 T cells are maintained and function in the post-septic host.

Our data collectively show CLP-induced sepsis results in a transient numerical reduction and long-term functional deficits of Ag-specific memory CD4 T cells, which contributes to the characteristic long-lasting immunoparalysis seen after sepsis and reduced protection to secondary infection. Secondary infection after sepsis, acquired while in the hospital or after discharge, is a leading cause of sepsis mortality (81–84). Lungs, blood stream, surgical site/soft tissue, and urinary tract are the most sites of secondary infection in septic patients, with *Pseudomonas* spp., *Staphylococcus* spp., *Candida albicans, E. coli*, and *Enterococcus* spp. being common secondary infection microbes (85). We recognize that the experimental pathogens used in this study (to establish either the primary or secondary infection) are not the “typical” pathogens found in sepsis patients. We chose to use *Listeria* and *Salmonella* for the following reasons: (1) both are well-established model pathogens for examining the immune response to bacteria, where attenuated strains are available for generating memory T cells and virulent strains are available to assess the protective capacity of the memory T cells; (2) *Listeria* and *Salmonella* infection stimulates a robust Ag-specific CD4 T cell response because the bacteria replicate in the phagosomes of dendritic cells and macrophages—the location of peptide:MHC II complex formation; and (3) the recombinant *Listeria* and *Salmonella* strains express known I-A^b^-restricted epitopes. Future studies could include engineering recombinant *P. aeruginosa, S. aureus*, and/or *S. pneumonia* to express the 2W1S (or some other) epitope that will enable testing of the 2W1S-specific memory CD4 T cell response in CLP-treated mice given a secondary infection with a clinically-relevant pathogen after recovery from the initial septic event. It is also important to note that while our study exclusively examined the *in vivo* function of memory CD4 T cells after sepsis, other publications have shown the environment is also a critical factor in sepsis-induced suppression of T cells (86). Following sepsis, a number of CD4 T cell extrinsic factors have been found to suppress the activity of CD4 T cells, including the reduced APC function and TNF signaling (79, 86). Sepsis also leads to dysfunction of the innate immune system, including impaired bacterial clearance by neutrophils, leading to increased susceptibility to *P. aeruginosa* and *S. aureus* (51, 87). Future studies could investigate the extent to which sepsis impairs innate immune responses, especially those related to trained innate immunity, in microbially-experienced mice containing a high frequency of Ag-experienced memory T cells.

In summary, our experiments have uncovered differences in recovery and function within the endogenous memory CD4 T cell compartment compared to what has been detected at the “bulk” CD4 T cell level. The data presented here will serve as the foundation for a number of future studies examining the behavior of endogenous, Ag-experienced memory CD4 T cells in the septic host, as well as methods of reversing the immunoparalysis typically observed within this population of immune cells vital to overall immune system fitness.

## Data Availability Statement

All datasets generated for this study are included in the article/supplementary material.

## Ethics Statement

This animal study was reviewed and approved by University of Minnesota Institutional Animal Care and Use Committees.

## Author Contributions

FS, TK, TD, WS, CD, JC-P, KM, and TG performed experiments and analyzed data. VB and TG provided input on the research design. FS, VB, and TG wrote and edited the manuscript. All authors read and approved the submitted version.

## Conflict of Interest

The authors declare that the research was conducted in the absence of any commercial or financial relationships that could be construed as a potential conflict of interest.

## References

[1] Shankar-Hari M Phillips GS Levy ML Seymour CW Liu VX Deutschman CS Developing a new definition and assessing new clinical criteria for septic shock: for the third international consensus definitions for sepsis and septic shock (Sepsis-3). JAMA. (2016) 315:775–87. 10.1001/jama.2016.0289 26903336 PMC4910392

[2] Brun-Buisson C Doyon F Carlet J Dellamonica P Gouin F Lepoutre A. Incidence, risk factors, and outcome of severe sepsis and septic shock in adults. A multicenter prospective study in intensive care units French ICU Group for Severe Sepsis. JAMA. (1995) 274:968–74. 10.1001/jama.1995.03530120060042 7674528

[3] Levy MM Fink MP Marshall JC Abraham E Angus D Cook D. 2001 SCCM/ESICM/ACCP/ATS/SIS international sepsis definitions conference. Intensive Care Med. (2001) 29:530–8. 10.1007/s00134-003-1662-x 12664219

[4] Hotchkiss RS Nicholson DW. Apoptosis and caspases regulate death and inflammation in sepsis. Nat Rev Immunol. (2006) 6:813–22. 10.1038/nri1943 17039247

[5] Rittirsch D Flierl MA Ward PA. Harmful molecular mechanisms in sepsis. Nat Rev Immunol. (2008) 8:776–87. 10.1038/nri2402 18802444 PMC2786961

[6] Hotchkiss RS Monneret G Payen D. Sepsis-induced immunosuppression: from cellular dysfunctions to immunotherapy. Nat Rev Immunol. (2013) 13:862–74. 10.1038/nri3552 24232462 PMC4077177

[7] Sasser SM Varghese M Joshipura M Kellermann A. Preventing death and disability through the timely provision of prehospital trauma care. Bull World Health Organ. (2006) 84:507. 10.2471/BLT.06.033605 16878215 PMC2627394

[8] Probst C Pape HC Hildebrand F Regel G Mahlke L Giannoudis P. 30 years of polytrauma care: an analysis of the change in strategies and results of 4849 cases treated at a single institution. Injury. (2009) 40:77–83. 10.1016/j.injury.2008.10.004 19117558

[9] Probst C Zelle BA Sittaro NA Lohse R Krettek C Pape HC. Late death after multiple severe trauma: when does it occur and what are the causes? J Trauma. (2009) 66:1212–7. 10.1097/TA.0b013e318197b97c 19359940

[10] Gentile LF Cuenca AG Efron PA Ang D Bihorac A Mckinley BA. Persistent inflammation and immunosuppression: a common syndrome and new horizon for surgical intensive care. J Trauma Acute Care Surg. (2012) 72:1491–501. 10.1097/TA.0b013e318256e000 22695412 PMC3705923

[11] Hotchkiss RS Monneret G Payen D. Immunosuppression in sepsis: a novel understanding of the disorder and a new therapeutic approach. Lancet Infect Dis. (2013) 13:260–8. 10.1016/S1473-3099(13)70001-X 23427891 PMC3798159

[12] Jensen IJ Sjaastad FV Griffith TS Badovinac VP. Sepsis-induced T cell immunoparalysis: the ins and outs of impaired T cell immunity. J Immunol. (2018) 200:1543–53. 10.4049/jimmunol.1701618 29463691 PMC5826615

[13] Hotchkiss RS Tinsley KW Swanson PE Schmieg RE Jr Hui JJ Chang KC. Sepsis-induced apoptosis causes progressive profound depletion of B and CD4+ T lymphocytes in humans. J Immunol. (2001) 166:6952–63. 10.4049/jimmunol.166.11.6952 11359857

[14] Unsinger J Kazama H Mcdonough JS Hotchkiss RS Ferguson TA. Differential lymphopenia-induced homeostatic proliferation for CD4+ and CD8+ T cells following septic injury. J Leukoc Biol. (2009) 85:382–90. 10.1189/jlb.0808491 19088177 PMC2653946

[15] Pepper M Jenkins MK. Origins of CD4(+) effector and central memory T cells. Nat Immunol. (2011) 12:467–71. 10.1038/ni.2038 21739668 PMC4212218

[16] Kaech SM Wherry EJ Ahmed R. Effector and memory T-cell differentiation: implications for vaccine development. Nat Rev Immunol. (2002) 2:251–62. 10.1038/nri778 12001996

[17] Harty JT Badovinac VP Shaping and reshaping CD8+ T-cell memory. Nat Rev Immunol. (2008) 8:107–19. 10.1038/nri2251 18219309

[18] Schenkel JM Masopust D. Tissue-resident memory T cells. Immunity. (2014) 41:886–97. 10.1016/j.immuni.2014.12.007 25526304 PMC4276131

[19] Beura LK Fares-Frederickson NJ Steinert EM Scott MC Thompson EA Fraser KA. CD4(+) resident memory T cells dominate immunosurveillance and orchestrate local recall responses. J Exp Med. (2019) 216:1214–29. 10.1084/jem.20181365 30923043 PMC6504216

[20] Taylor JJ Jenkins MK CD4+ memory T cell survival. Curr Opin Immunol. (2011) 23:319–23. 10.1016/j.coi.2011.03.010 21524898

[21] Condotta SA Cabrera-Perez J Badovinac VP Griffith TS. T-cell-mediated immunity and the role of TRAIL in sepsis-induced immunosuppression. Crit Rev Immunol. (2013) 33:23–40. 10.1615/CritRevImmunol.2013006721 23510024 PMC3625932

[22] Cabrera-Perez J Condotta SA Badovinac VP Griffith TS. Impact of sepsis on CD4 T cell immunity. J Leukoc Biol. (2014) 96:767–77. 10.1189/jlb.5MR0114-067R 24791959 PMC4197564

[23] Beura LK Hamilton SE Bi K Schenkel JM Odumade OA Casey KA. Normalizing the environment recapitulates adult human immune traits in laboratory mice. Nature. (2016) 532:512–6. 10.1038/nature17655 27096360 PMC4871315

[24] Huggins MA Sjaastad FV Pierson M Kucaba TA Swanson W Staley C. Microbial exposure enhances immunity to pathogens recognized by TLR2 but increases susceptibility to cytokine storm through TLR4 sensitization. Cell Rep. (2019) 28:1729–43.e5. 10.1016/j.celrep.2019.07.028 31412243 PMC6703181

[25] Rittirsch D Huber-Lang MS Flierl MA Ward PA. Immunodesign of experimental sepsis by cecal ligation and puncture. Nat Protoc. (2009) 4:31–6. 10.1038/nprot.2008.214 19131954 PMC2754226

[26] Duong S Condotta SA Rai D Martin MD Griffith TS Badovinac VP. Polymicrobial sepsis alters antigen-dependent and -independent memory CD8 T cell functions. J Immunol. (2014) 192:3618–25. 10.4049/jimmunol.1303460 24646738 PMC4001259

[27] Cabrera-Perez J Babcock JC Dileepan T Murphy KA Kucaba TA Badovinac VP. Gut microbial membership modulates CD4 T cell reconstitution and function after sepsis. J Immunol. (2016) 197:1692–8. 10.4049/jimmunol.1600940 27448587 PMC4992581

[28] Goldberg MF Roeske EK Ward LN Pengo T Dileepan T Kotov DI. Salmonella persist in activated macrophages in T cell-sparse granulomas but are contained by surrounding CXCR3 ligand-positioned Th1 cells. Immunity. (2018) 49:1090–102.e7. 10.1016/j.immuni.2018.10.009 30552021 PMC6301113

[29] Moon JJ Chu HH Pepper M Mcsorley SJ Jameson SC Kedl RM. Naive CD4(+) T cell frequency varies for different epitopes and predicts repertoire diversity and response magnitude. Immunity. (2007) 27:203–13. 10.1016/j.immuni.2007.07.007 17707129 PMC2200089

[30] Moon JJ Chu HH Hataye J Pagan AJ Pepper M Mclachlan JB. Tracking epitope-specific T cells. Nat Protoc. (2009) 4:565–81. 10.1038/nprot.2009.9 19373228 PMC3517879

[31] Pepper M Pagan AJ Igyarto BZ Taylor JJ Jenkins MK. Opposing signals from the Bcl6 transcription factor and the interleukin-2 receptor generate T helper 1 central and effector memory cells. Immunity. (2011) 35:583–95. 10.1016/j.immuni.2011.09.009 22018468 PMC3208313

[32] Pagan AJ Pepper M Chu HH Green JM Jenkins MK. CD28 promotes CD4+ T cell clonal expansion during infection independently of its YMNM and PYAP motifs. J Immunol. (2012) 189:2909–17. 10.4049/jimmunol.1103231 22896637 PMC3464098

[33] Nelson RW Mclachlan JB Kurtz JR Jenkins MK. CD4+ T cell persistence and function after infection are maintained by low-level peptide:MHC class II presentation. J Immunol. (2013) 190:2828–34. 10.4049/jimmunol.1202183 23382562 PMC3594488

[34] Cabrera-Perez J Condotta SA James BR Kashem SW Brincks EL Rai D. Alterations in antigen-specific naive CD4 T cell precursors after sepsis impairs their responsiveness to pathogen challenge. J Immunol. (2015) 194:1609–20. 10.4049/jimmunol.1401711 25595784 PMC4412277

[35] Meakins JL Pietsch JB Bubenick O Kelly R Rode H Gordon J. Delayed hypersensitivity: indicator of acquired failure of host defenses in sepsis and trauma. Ann Surg. (1977) 186:241–50. 10.1097/00000658-197709000-00002 142452 PMC1396336

[36] Brown R Bancewicz J Hamid J Patel NJ Ward CA Farrand RJ. Failure of delayed hypersensitivity skin testing to predict postoperative sepsis and mortality. Br Med J. (1982) 284:851–3. 10.1136/bmj.284.6319.851 6802324 PMC1496294

[37] Meakins JL Christou NV Bohnen J Maclean LD. Failure of delayed hypersensitivity skin testing to predict postoperative sepsis and mortality. Br Med J. (1982) 285:1207–8. 10.1136/bmj.285.6349.1207-a 6812812 PMC1500143

[38] Vissinga C Nagelkerken L Zijlstra J Hertogh-Huijbregts A Boersma W Rozing J. A decreased functional capacity of CD4+ T cells underlies the impaired DTH reactivity in old mice. Mech Ageing Dev. (1990) 53:127–39. 10.1016/0047-6374(90)90065-N 1971315

[39] Gurung P Rai D Condotta SA Babcock JC Badovinac VP Griffith TS. Immune unresponsiveness to secondary heterologous bacterial infection after sepsis induction is TRAIL dependent. J Immunol. (2011) 187:2148–54. 10.4049/jimmunol.1101180 21788440 PMC3159846

[40] Condotta SA Rai D James BR Griffith TS Badovinac VP. Sustained and incomplete recovery of naive CD8+ T cell precursors after sepsis contributes to impaired CD8+ T cell responses to infection. J Immunol. (2013) 190:1991–2000. 10.4049/jimmunol.1202379 23355736 PMC3578009

[41] Condotta SA Khan SH Rai D Griffith TS Badovinac VP. Polymicrobial sepsis increases susceptibility to chronic viral infection and exacerbates CD8+ T cell exhaustion. J Immunol. (2015) 195:116–25. 10.4049/jimmunol.1402473 25980007 PMC4475506

[42] Sjaastad FV Condotta SA Kotov JA Pape KA Dail C Danahy DB. Polymicrobial sepsis chronic immunoparalysis is defined by diminished Ag-specific T cell-dependent B cell responses. Front Immunol. (2018) 9:2532. 10.3389/fimmu.2018.02532 30429857 PMC6220049

[43] Mcdermott DS Varga SM. Quantifying antigen-specific CD4 T cells during a viral infection: CD4 T cell responses are larger than we think. J Immunol. (2011) 187:5568–76. 10.4049/jimmunol.1102104 22043009 PMC3221938

[44] Kotov DI Kotov JA Goldberg MF Jenkins MK. Many Th cell subsets have fas ligand-dependent cytotoxic potential. J Immunol. (2018) 200:2004–12. 10.4049/jimmunol.1700420 29436413 PMC5840022

[45] Benoun JM Peres NG Wang N Pham OH Rudisill VL Fogassy ZN. Optimal protection against Salmonella infection requires noncirculating memory. Proc Natl Acad Sci USA. (2018) 115:10416–21. 10.1073/pnas.1808339115 30254173 PMC6187142

[46] Hess J Ladel C Miko D Kaufmann SH. Salmonella typhimurium aroA- infection in gene-targeted immunodeficient mice: major role of CD4+ TCR-alpha beta cells and IFN-gamma in bacterial clearance independent of intracellular location. J Immunol. (1996) 156:3321–6. 8617956

[47] Monack DM Mueller A Falkow S. Persistent bacterial infections: the interface of the pathogen and the host immune system. Nat Rev Microbiol. (2004) 2:747–65. 10.1038/nrmicro955 15372085

[48] Johanns TM Ertelt JM Lai JC Rowe JH Avant RA Way SS. Naturally occurring altered peptide ligands control Salmonella-specific CD4+ T cell proliferation, IFN-gamma production, and protective potency. J Immunol. (2010) 184:869–76. 10.4049/jimmunol.0901804 20026741 PMC2867337

[49] Johanns TM Ertelt JM Rowe JH Way SS. Regulatory T cell suppressive potency dictates the balance between bacterial proliferation and clearance during persistent Salmonella infection. PLoS Pathog. (2010) 6:e1001043. 10.1371/journal.ppat.1001043 20714351 PMC2920851

[50] Marzo AL Vezys V Williams K Tough DF Lefrancois L. Tissue-level regulation of Th1 and Th2 primary and memory CD4 T cells in response to Listeria infection. J Immunol. (2002) 168:4504–10. 10.4049/jimmunol.168.9.4504 11970995

[51] Delano MJ Thayer T Gabrilovich S Kelly-Scumpia KM Winfield RD Scumpia PO. Sepsis induces early alterations in innate immunity that impact mortality to secondary infection. J Immunol. (2011) 186:195–202. 10.4049/jimmunol.1002104 21106855 PMC3771366

[52] Condotta SA Richer MJ Badovinac VP Harty JT. Probing CD8 T cell responses with *Listeria monocytogenes* infection. Adv Immunol. (2012) 113:51–80. 10.1016/B978-0-12-394590-7.00005-1 22244579

[53] Brocke S Hahn H. Heat-killed *Listeria monocytogenes* and *L. monocytogenes* soluble antigen induce clonable CD4+ T lymphocytes with protective and chemotactic activities *in vivo*. Infect. Immun. (1991) 59:4531–9. 10.1128/IAI.59.12.4531-4539.1991 1682264 PMC259074

[54] Harty JT Schreiber RD Bevan MJ. CD8 T cells can protect against an intracellular bacterium in an interferon gamma-independent fashion. Proc Natl Acad Sci USA. (1992) 89:11612–6. 10.1073/pnas.89.23.11612 1360672 PMC50603

[55] Bhardwaj V Kanagawa O Swanson PE Unanue ER Chronic Listeria infection in SCID mice: requirements for the carrier state and the dual role of T cells in transferring protection or suppression. J Immunol. (1998) 160:376–84. 9551994

[56] Vincent JL Marshall JC Namendys-Silva SA Francois B Martin-Loeches I Lipman J. Assessment of the worldwide burden of critical illness: the intensive care over nations (ICON) audit. Lancet Respir Med. (2014) 2:380–6. 10.1016/S2213-2600(14)70061-X 24740011

[57] Cohen J Vincent JL Adhikari NK Machado FR Angus DC Calandra T. Sepsis: a roadmap for future research. Lancet Infect Dis. (2015) 15:581–614. 10.1016/S1473-3099(15)70112-X 25932591

[58] Le Gros G Ben-Sasson SZ Seder R Finkelman FD Paul WE. Generation of interleukin 4 (IL-4)-producing cells *in vivo* and *in vitro*: IL-2 and IL-4 are required for in vitro generation of IL-4-producing cells. J Exp Med. (1990) 172:921–9. 10.1084/jem.172.3.921 2117636 PMC2188542

[59] Hsieh CS Heimberger AB Gold JS O'garra A Murphy KM. Differential regulation of T helper phenotype development by interleukins 4 and 10 in an alpha beta T-cell-receptor transgenic system. Proc Natl Acad Sci USA. (1992) 89:6065–9. 10.1073/pnas.89.13.6065 1385868 PMC49438

[60] Chen X Ye J Ye J. Analysis of peripheral blood lymphocyte subsets and prognosis in patients with septic shock. Microbiol Immunol. (2011) 55:736–42. 10.1111/j.1348-0421.2011.00373.x 21831206

[61] Gouel-Cheron A Venet F Allaouchiche B Monneret G. CD4+ T-lymphocyte alterations in trauma patients. Crit Care. (2012) 16:432. 10.1186/cc11376 22734607 PMC3580654

[62] Inoue S Suzuki-Utsunomiya K Okada Y Taira T Iida Y Miura N. Reduction of immunocompetent T cells followed by prolonged lymphopenia in severe sepsis in the elderly. Crit Care Med. (2013) 41:810–9. 10.1097/CCM.0b013e318274645f 23328259

[63] Baker CC Miller CL Trunkey DD Lim RC Jr. Identity of mononuclear cells which compromise the resistance of trauma patients. J Surg Res. (1979) 26:478–87. 10.1016/0022-4804(79)90037-4 312363

[64] Hansbrough JF Bender EM Zapata-Sirvent R Anderson J. Altered helper and suppressor lymphocyte populations in surgical patients. A measure of postoperative immunosuppression. Am J Surg. (1984) 148:303–7. 10.1016/0002-9610(84)90459-8 6236703

[65] Uzzaman A Cho SH. Chapter 28: Classification of hypersensitivity reactions. Allergy Asthma Proc. (2012) 33(Suppl. 1):S96–99. 10.2500/aap.2012.33.3561 22794701

[66] Hamilton SE Badovinac VP Beura LK Pierson M Jameson SC Masopust D. New insights into the immune system using dirty mice. J Immunol. (2020) 205:3–11. 10.4049/jimmunol.2000171 32571979 PMC7316151

[67] Grayson JM Harrington LE Lanier JG Wherry EJ Ahmed R. Differential sensitivity of naive and memory CD8+ T cells to apoptosis *in vivo*. J Immunol. (2002) 169:3760–70. 10.4049/jimmunol.169.7.3760 12244170

[68] Hotchkiss RS Osmon SB Chang KC Wagner TH Coopersmith CM Karl IE. Accelerated lymphocyte death in sepsis occurs by both the death receptor and mitochondrial pathways. J Immunol. (2005) 174:5110–8. 10.4049/jimmunol.174.8.5110 15814742

[69] Unsinger J Mcglynn M Kasten KR Hoekzema AS Watanabe E Muenzer JT. IL-7 promotes T cell viability, trafficking, and functionality and improves survival in sepsis. J Immunol. (2010) 184:3768–79. 10.4049/jimmunol.0903151 20200277 PMC2914630

[70] Serbanescu MA Ramonell KM Hadley A Margoles LM Mittal R Lyons JD. Attrition of memory CD8 T cells during sepsis requires LFA-1. J Leukoc Biol. (2016) 100:1167–80. 10.1189/jlb.4A1215-563RR 27286793 PMC5069090

[71] Danahy DB Anthony SM Jensen IJ Hartwig SM Shan Q Xue HH. Polymicrobial sepsis impairs bystander recruitment of effector cells to infected skin despite optimal sensing and alarming function of skin resident memory CD8 T cells. PLoS Pathog. (2017) 13:e1006569. 10.1371/journal.ppat.1006569 28910403 PMC5599054

[72] Danahy DB Strother RK Badovinac VP Griffith TS. Clinical and experimental sepsis impairs CD8 T-cell-mediated immunity. Crit Rev Immunol. (2016) 36:57–74. 10.1615/CritRevImmunol.2016017098 27480902 PMC5314458

[73] Xie J Chen CW Sun Y Laurie SJ Zhang W Otani S. Increased attrition of memory T cells during sepsis requires 2B4. JCI Insight. (2019) 4:e126030. 10.1172/jci.insight.126030 31045575 PMC6538360

[74] Hotchkiss RS Swanson PE Knudson CM Chang KC Cobb JP Osborne DF. Overexpression of Bcl-2 in transgenic mice decreases apoptosis and improves survival in sepsis. J Immunol. (1999) 162:4148–56. 10.1097/00024382-199806001-00219 10201940

[75] Hotchkiss RS Chang KC Swanson PE Tinsley KW Hui JJ Klender P. Caspase inhibitors improve survival in sepsis: a critical role of the lymphocyte. Nat Immunol. (2000) 1:496–501. 10.1038/82741 11101871

[76] Inoue S Unsinger J Davis CG Muenzer JT Ferguson TA Chang K. IL-15 prevents apoptosis, reverses innate and adaptive immune dysfunction, and improves survival in sepsis. J Immunol. (2010) 184:1401–9. 10.4049/jimmunol.0902307 20026737 PMC2937828

[77] Unsinger J Kazama H Mcdonough JS Griffith TS Hotchkiss RS Ferguson TA. Sepsis-induced apoptosis leads to active suppression of delayed-type hypersensitivity by CD8+ regulatory T cells through a TRAIL-dependent mechanism. J Immunol. (2010) 184:6766–72. 10.4049/jimmunol.0904054 20483771 PMC2887093

[78] Cavassani KA Carson WFT Moreira AP Wen H Schaller MA Ishii M. The post sepsis-induced expansion and enhanced function of regulatory T cells create an environment to potentiate tumor growth. Blood. (2010) 115:4403–11. 10.1182/blood-2009-09-241083 20130237 PMC2881495

[79] Stieglitz D Schmid T Chhabra NF Echtenacher B Mannel DN Mostbock S. TNF and regulatory T cells are critical for sepsis-induced suppression of T cells. Immun Inflamm Dis. (2015) 3:374–85. 10.1002/iid3.75 26734459 PMC4693718

[80] Rimmele T Payen D Cantaluppi V Marshall J Gomez H Gomez A. Immune cell phenotype and function in sepsis. Shock. (2016) 45:282–91. 10.1097/SHK.0000000000000495 26529661 PMC4752878

[81] Hall MW Knatz NL Vetterly C Tomarello S Wewers MD Volk HD. Immunoparalysis and nosocomial infection in children with multiple organ dysfunction syndrome. Intensive Care Med. (2011) 37:525–32. 10.1007/s00134-010-2088-x 21153402 PMC5224706

[82] Otto GP Sossdorf M Claus RA Rodel J Menge K Reinhart K. The late phase of sepsis is characterized by an increased microbiological burden and death rate. Crit Care. (2011) 15:R183. 10.1186/cc10332 21798063 PMC3387626

[83] Goldenberg NM Leligdowicz A Slutsky AS Friedrich JO Lee WL. Is nosocomial infection really the major cause of death in sepsis? Crit Care. (2014) 18:540. 10.1186/s13054-014-0540-y 25672933 PMC4331295

[84] Daviaud F Grimaldi D Dechartres A Charpentier J Geri G Marin N. Timing and causes of death in septic shock. Ann Intensive Care. (2015) 5:16. 10.1186/s13613-015-0058-8 26092499 PMC4474967

[85] Zhao GJ Li D Zhao Q Song JX Chen XR Hong GL. Incidence, risk factors and impact on outcomes of secondary infection in patients with septic shock: an 8-year retrospective study. Sci Rep. (2016) 6:38361. 10.1038/srep38361 27924831 PMC5141415

[86] Strother RK Danahy DB Kotov DI Kucaba TA Zacharias ZR Griffith TS. Polymicrobial sepsis diminishes dendritic cell numbers and function directly contributing to impaired primary CD8 T cell responses *in vivo*. J Immunol. (2016) 197:4301–11. 10.4049/jimmunol.1601463 27798171 PMC5123856

[87] Robertson CM Perrone EE Mcconnell KW Dunne WM Boody B Brahmbhatt T. Neutrophil depletion causes a fatal defect in murine pulmonary *Staphylococcus aureus* clearance. J Surg Res. (2008) 150:278–85. 10.1016/j.jss.2008.02.009 18621398 PMC2605623

